# *Lactococcus Lactis* Strain A5 Producing Nisin-like Bacteriocin Active against Gram Positive and Negative Bacteria

**DOI:** 10.21315/tlsr2017.28.2.8

**Published:** 2017-07-31

**Authors:** Nur Shazana Azhar, Noor Hasniza Md Zin, Tengku Haziyamin Tengku Abdul Hamid

**Affiliations:** Department of Biotechnology, Kulliyyah of Science, International Islamic University Malaysia 25200 Kuantan, Pahang

**Keywords:** LAB, Probiotic, Nisin, Bacteriocin, *Lactococcus lactis*

## Abstract

In this study, a Lactic acid bacteria (LAB) strain was isolated on MRS medium from gastro-intestinal tissues of Broadhead catfish (*Clarias macrocephalus*). Out of 50 isolates, 25 isolates were found to be positive on lactose utilisation test and were identified to be gram positive cocci. Using disc diffusion methods, one out of 22 isolates, i.e., a strain A5 demonstrated inhibitions against three indicator organisms; *Bacillus cereus*, *Staphylococcus aureus* and *Salmonella thyphimurium*. Partial 16S rDNA sequencing identified isolate A5 as a member of *Lactococcus lactis*, with 100% DNA homology. Cell free supernatant fluid from *Lactococcus lactis* A5 showed inhibitory activities against both gram positive pathogens (*Bacillus cereus* and *Staphylococcus aureus*) and gram negative pathogens (*Salmonella thyphimurium*). Chloroform precipitated bacteriocin retained antagonistic activities in the presence of catalase and lysozyme; and was completely inactivated by Proteinase K treatment. The bacteriocin has a molecular weight of 3.4 kDa, based on SDS-PAGE analysis and the extract was heat stable at 37°C and 65°C, for 15 minutes. The antibacterial activity was suppressed with the addition of EDTA but was significantly increased with the addition of SDS, Triton X-100, Tween 20 and Tween 80. This bacteriocin belongs to class 1 bacteriocin, which was shown to have a nisin-like properties. This strain can be used as potential probiotics in animal or aquaculture feeding; and the bacteriocin it produces will be useful in food preservative.

## Abstract (ru)

Dalam kajian ini, strain bakteria asid Laktik (LAB) telah dipencilkan pada medium MRS daripada tisu gastro-intestinal ikan keli *Broadhead* (*Clarias macrocephalus*). Daripada 50 isolat, 25 isolat didapati positif pada ujian penggunaan laktosa dan dikenal pasti sebagai *cocci* gram positif. Dengan menggunakan kaedah penyebaran cakera, satu daripada 22 isolat, iaitu, strain A5 menunjukkan hambatan terhadap tiga organisma penunjuk; *Bacillus cereus*, *Staphylococcus aureus* dan *Salmonella thyphimurium*. Susunan seksyen 16S rDNA dikenal pasti mengasingkan A5 sebagai ahli *Lactococcus lactis*, dengan 100% homologi DNA. Cecair supernatan bebas dari *Lactococcus lactis* A5 menunjukkan aktiviti perencatan terhadap kedua-dua patogen positif gram (*Bacillus cereus* dan *Staphylococcus aureus*) dan gram negatif patogen (*Salmonella thyphimurium*). Chloroform precipitated bacteriocin mengekalkan aktiviti antagonistik di hadapan katalase dan lysozyme; Dan benar-benar tidak aktif oleh rawatan Proteinase K. Bacteriocin mempunyai berat molekul 3.4 kDa, berdasarkan analisis SDS-PAGE dan ekstrak adalah haba stabil pada 37 °C dan 65 °C, selama 15 minit. Aktiviti antibakteria direncat dengan penambahan EDTA tetapi meningkat dengan ketara dengan penambahan SDS, Triton X-100, Tween 20 dan Tween 80. Bacteriosin ini tergolong dalam kelas 1 bacteriocin, yang ditunjukkan mempunyai sifat seperti nisin. Strain ini boleh digunakan sebagai probiotik yang berpotensi dalam pemakanan haiwan atau akuakultur; dan bacteriosin yang dihasilkan akan berguna dalam pengawet makanan.

## INTRODUCTION

Driven by the increasing demand for animal protein and food security, aquaculture is a fast growing industry that becomes a vital economy in developing countries such as Malaysia. Intensive fish farming normally results in overcrowding and low water quality that promote diseases in fish. Ultimately, high mortality and costly treatment result in poor productivity and reduced marketability due to a less appealing fish appearances. Antibiotics have always been the treatment of choice in order to improve the growth and feed conversion in poultry and other animals (Shin *et al*. 2008). Overuse or misuse of antibiotics can potentially result in the emergence of resistant pathogens (Cabello 2006). There is also concern on the spread of antibiotic resistant gene from fish pathogen to that of humans (Korada & Yarla 2014). These have spurred the phenomenal search for alternative antimicrobial compounds through intensified natural product screening researches. Probiotic supplementation can reduce disease outbreaks by enhancing the immune system and increasing the feed efficiency of fish. The application of probiotics in aquaculture has been used as a mean to control disease, enhancing immune response, providing nutritional and enzymatic contributions to the digestion of the host and improving water quality (Korada & Yarla 2014; Qi *et al*. 2009; Rengpipat *et al*. 2000). Probiotics are live microbial feed supplements which improve the intestinal balance of the host microbiota (Fuller 1989), and the growth performance of larval or immature fish (Lara-Flores & Olvera-Novoa 2013). Probiotics could inhibit pathogenic organisms by producing antimicrobial substances such as hydrogen peroxide, organic acids, diacetyl, acetoin, reuterin, and bacteriocins (Rajaram *et al*. 2010). Antimicrobial compounds such as bacteriocin may become an alternative treatment in the prevention of bacterial diseases in fishes. Bacteriocins are ribosomally synthesised proteinaceous substances produced by bacteria that show antimicrobial activity against bacteria that is closely related to the producer strain (Trivedi *et al*. 2013). The fish gut was shown to be a natural habitat for probiotic microorganisms, as it is normally found in the digestive tracts of many fish species from different environments (Buntin *et al*. 2008).

Lactic acid bacteria (LAB) are a heterologous group of bacteria widespread in nature and they are commonly found in milk, dairy products, plant material, intestinal tracts of humans and animals (Utkarsha & Milind 2013). Major groups of probiotic bacteria are LAB; which generally consists of gram-positive, catalase positive, non-motile and non-sporulating; and it produces lactic acid as a major product from carbohydrate fermentation. Ideally, LAB used in feed additives should be derived from the microbiota of the targeted animals (Kosin & Rakshit 2006)). Catfish aquaculture is popular in Malaysia and a species *Clarias macrocephalus* or locally known as *Keli bunga* are common species (Abu *et al*. 2010). In this study, an LAB strain was isolated from the gut of *C. macrocephalus*; and the bacteriocin it produces was purified and then characterised. The antagonistic potential of this bacteriocin against pathogenic microorganisms was investigated, in an attempt to explore its future use in aquaculture and other applications.

## MATERIALS AND METHOD

### Isolation and Identification of LAB Strains

Digestive tracts from two catfishes (labeled as sample A1 and B1) were sterilely dissected and washed. The fish intestines were cut, weighed and homogenized in phosphate buffered saline (PBS) and vortexed. Samples were serially diluted with 0.85% NaCl, and were pour plated on MRS agar (Difco, USA). Following incubation at 30°C for 48 h, single colonies were picked out from the agar plates (10^−6^ dilution), sub-cultured onto MRS agar and further incubated at 30°C for 24 h to 48 h, until colonies became visible. Isolated colonies were inoculated into the MRS broth, incubated at 30°C for 24 to 48 h to reach 1 × 10^7^ CFU/ml, and kept at −76°C under 15%(w/v) glycerol. The isolates were identified according to their morphological and biochemical characteristics by using gram staining, lactose utilisation, catalase test and sugar fermentation test using API 50 CHL strips (Biomerieux, France). The genomic extractions were also carried out for rDNA gene sequencing.

For lactose utilisation test, the selected colonies were streaked onto lactose containing nutrient agar (NA) with of 0.005 g/L bromocresol purple as pH indicator dye. The plates were incubated at 30°C for 24 h. Isolates that were able to utilise lactose and produce acid were differentiated by the change in the media colour from violet to yellow. Catalase activity was tested by adding a drop of 30% hydrogen peroxide solution onto the cell smears. Only isolates that showed negative reaction were subjected to further tests for identification.

To test for antimicrobial activity, the disc diffusion method was employed. *Bacillus cereus* ATCC 11778, *Staphylococcus aureus* ATCC 33592, *Salmonella thyphimurium* S1000, *Escherichia coli* ATCC 25922 and *Pseudomonas aeruginosa* ATCC 27853 were used as indicator microorganisms (retrieved from Institute Medical Research, IMR, Kuala Lumpur). Potential probiotic LAB strains were propagated in the MRS broth medium and incubated at 30°C for 24 h until cell density of 1 × 10^7^ CFU/mL were reached. Sterile blank paper discs (Oxoid), were dipped into approximately 500 μl broth, air dried and placed unto plates pre-streaked with indicator organisms. The indicator cells were cultured overnight in Muller-Hinton (MH) broth. About 100 μl of these cultures (adjusted to approximately 1 × 10^6^ CFU/ml ≈ 0.1 OD_600nm_) were spread over MH agar plates, in triplicates. The plates were incubated at 37°C for 24 h and the inhibition zones formed surrounding the discs were observed. The inhibition activities were measured based on minimal inhibition of two fold dilution series of samples.

Fermentation test on the selected strain was carried out using API 50 CHL kit (Biomerieux, France) and for rDNA sequence analysis, the genomic DNA was extracted using QIAamp DNA mini kit (Qiagen, USA). The 16S rDNA gene fragments of ~1.5 kb were amplified using a pair of universal primers 27F and 1492 R (forward: 5′-AGA GTT TGA TCC TGG CTC AG -3′ and reverse: 5′-GGT TAC CTT GTT ACG ACT T-3′). Amplification was carried out in a total reaction volume of 50 μl, using DNA amplification kit (Vivantis, Malaysia). Reaction mixtures contained about 250ng DNA template, 1 μl of each forward and reverse primers (15 pmoles each) and 25 μl of 2X Taq Master mix (Vivantis, Malaysia). The 2X Taq Master mix contained Taq DNA polymerase at 0.05 Uml^−1^, 2X Vi buffer A, 0.4 mM dNTPs and 3.0 mM MgCl_2_. The amplifications were performed with initial denaturation at 94°C for 4 min, 29 cycles of denaturation (94°C for 2 min); annealing (55°C for 1 min); and final extension (at 72°C for 10 min) followed by a final holding at 4°C for 1 minute. Finally, 5.0 μl PCR products were subjected to (1.0 %) agarose gel electrophoresis. The gel was stained with ethidium bromide and analysed using a gel imager (Alpha Imager). The 16S rDNA sequencing was carried out at a sequencing agency (First BASE Laboratories Sdn. Bhd., Malaysia). The sequences were analysed using Multialign tool software. The Basic Local Alignment Search Tool Nucleotide (BLASTN) search tool was available online at National Centre for Biotechnology Information (NCBI) and accessible at http://www.ncbi.nlm.nih.gov. A phylogenetic tree was also generated using Software MEGA 2.1, using neighbour joining method in which the *Staphyloccocus aureus* gene was used as an out group.

### Extraction of Bacteriocin

Solvent extraction method using chloroform was used in order to purify bacteriocin (Burianek & Yousef 2000). About 15 mL of chloroform was mixed with 30 mL of cell-free culture supernatant (at aqueous-solvent ratio 2:1) and this was vigorously shaken at 400 rpm for 20 min for mixing. Following centrifugation at 9000 rpm for 30 min at 18°C, the top aqueous layer was discarded and the interfacial precipitate was retained. The pellet was dried to eliminate residual chloroform in a speed vacuum and suspended in 0.85% NaCl. The recovered protein was then checked for antibacterial activity using the agar disk diffusion method, and quantified by using Bradford protein assay (Bradford 1976). The purified bacteriocin was subjected to other characterisations, and visualised using SDS-PAGE (Laemmli 1970).

### Antagonistic Assay

The antibacterial spectrum of bacteriocin producing LAB was determined using the spot-on-lawn method (Barefoot & Klaenhammer 1983). Sterile paper discs (Oxoid) were impregnated with 50μl of the extracted bacteriocin. The paper discs containing bacteriocin were briefly air dried and layered on the MH agar of which the surface was pre-streaked with 10μl overnight culture of indicator organism, either *Bacillus cereus* or *Staphylococcus aureus*; or *Salmonella thyphimurium*. Each plate was done in triplicate and for negative control, 50 μl of MRS broth and chloroform (1:1) was used instead of the sample. The plates were incubated for 18 h at 37°C, and the diameter of inhibition zones were measured.

### Effects of Enzyme, EDTA, Detergents and Temperature on Antimicrobial Activity

Enzymes used in this study were proteinase K (Oxoid), lysozyme (Oxoid) and catalase (Sigma), at 0.5 mgml^−1^ concentration. All preparations were incubated at 37°C for 2 h. The effects of EDTA and some detergents (SDS, Triton X-100, Tween 20 and Tween 80) on antimicrobial activities were also determined. The purified bacteriocin was incubated at 37°C for 2 h with SDS, EDTA, Triton X-100, Tween 20 and Tween 80 at concentrations of 1% and 8%. Using agar diffusion test, negative controls were also carried out containing these reagents without the bacteriocin extract. Sensitivity of bacteriocin to heat was verified by heating the bacteriocin produced by *L. lactis* A5 for 15 min at 37°C, 65°C, 100°C and 121°C. The disc diffusion assay was performed to detect activities against test organisms. After each treatment, both sample and control were tested for antimicrobial activities against *S. thyphimurium* by the disc diffusion assay as described above. Untreated extract containing bacteriocin was used as control.

## RESULTS AND DISCUSSION

### Screening for Lactic Acid Bacteria

**Figure 1:**
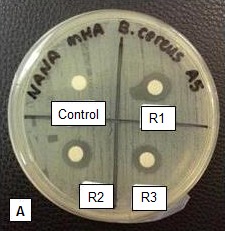

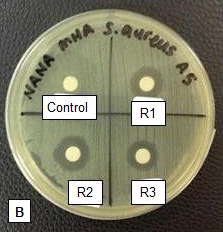

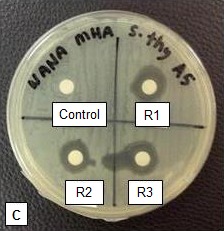

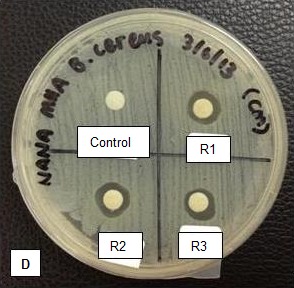

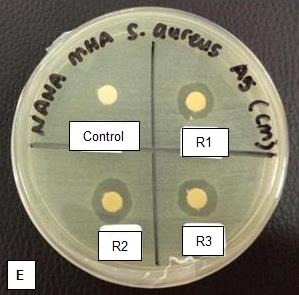

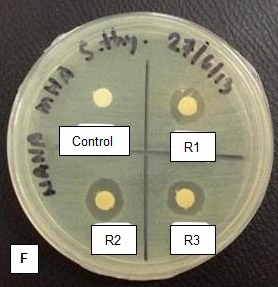
Antagonistic activities of isolate A5 crude supernatant against indicator microorganisms Panel A:*Bacillus cereus* (Panel A); *Staphylococcus aureus* (Panel B*)*; *Salmonella thyphimurium* (Panel C); and each with a negative control (0.85% NaCl). The R1, R2 and R3 represent the replicates. Antibacterial activities of purified bacteriocin extract (using chloroform) towards *Bacillus cereus* (Panel D), *Staphylococcus aureus* (Panel E) and *Salmonella thyphimurium* (Panel F). Control was a mixture of MRS broth and chloroform. Labels R1, R2 and R3 represents the replicates.

About 50 isolates were initially selected from two catfish samples; A1 and B1. Out of the 50 isolates, 22 isolates (11 from sample A1 and 11 from sample B1) were found to be positive towards the lactose utilisation test and showed to be catalase negative. Most of these colonies appeared as convex with entire margin, milky white circular in shape, found to be non-motile gram positive cocci and non-spore formers. Out of these, only one isolate, e.g. A5, demonstrated a broad spectrum inhibition against gram positive (*Bacillus cereus*, *Staphylococcus aureus*) and gram negative (*Salmonella thyphimurium*) pathogens (see Fig. 1, panel A, B and C). Other gram negative organism showed no inhibition. This isolate was selected further for characterisation analysis. Based on sugar fermentation tests, this isolate was able to ferment L-arabinose, D-xylose, galactose, glucose, fructose, mannose, N-acetyl-glucosamine, amygladin, arbutin, salicin, cellobiose, maltose, lactose, trehalose, starch and gentiobiose. This fermentation profile was consistent with that of genus *Lactococcus.* From various aquaculture species, other than *Lactococcus*, the isolations of *Bacillus, Enterococcus, Lactobacilus, Pediococcus, Leuconostoc* and *Carnobacteria* were also reported (Ringø & Gatesoupe 1998; Buntin *et al*., 2008 and Mohapatra *et al*., 2012).

**Figure 2:**
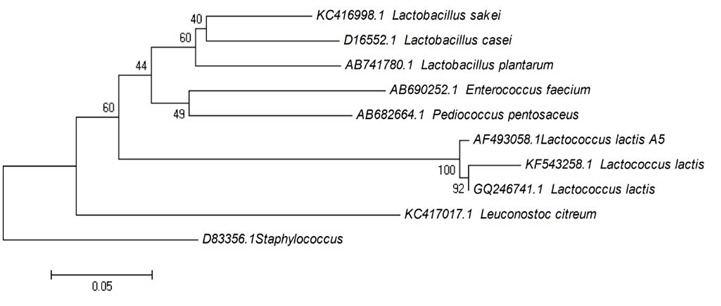
The phylogenetic tree of 16S rRNA gene sequences for *Lactococcus lactis* A5 (KP064393) and other closely related lactic acid bacteria. The tree generated using on Neighbor-joining with scale bar equals 0.05 substitutions per nucleotide. Bootstrap values (%) are indicated at the branches from 1000 replications. *Staphylococcus aureus*. was used as an outgroup.

PCR amplification of the 16S rDNA gene from isolate A5 resulted in the amplification of 1.5kb product of an expected size rDNA gene. The 16S rDNA sequence was assigned with an accession number of KP064393 at NCBI Genebank database. Result from BLASTN search revealed a high similarity (~100%) of this strain to other *Lactococcus lactis* strains. This result was in agreement with the morphological and fermentation profile studies carried out above. As shown in Figure 2, a phylogenetic tree was then constructed with other LAB strains of *Lactococcus* sp. and *Lactobacillus* sp.

On antagonistic test, isolate A5 showed zones of inhibition against *Bacillus cereus, Staphylococcus aureus* and *Salmonella thyphimurium*; at 12.0 mm, 14.0 mm and 14.0 mm inhibitory diameter, respectively (Fig. 1). Antagonism against closely related gram positive bacteria is a common feature of LAB. Meanwhile, the ability to antagonise gram negative strains were reported, however these were not as common. The antimicrobial action of LAB is mainly due to various factors including the production of metabolites such as organic acids (lactic and acetic acid), hydrogen peroxide, ethanol, diacetyl, acetaldehyde, acetoine, carbon dioxide, reuterin, reutericyclin and bacteriocins (Šušković *et al*. 2011).

### Characterisation of Bacteriocin Extract

The purified fraction of bacteriocin produced a zone of inhibition of sizes between 12.0 to 14.0 mm diameter against indicator bacteria (*Bacillus cereus*, *Staphylococcus aureus* and *Salmonella thyphimurium*), as shown in Figure 1 (Panel D, E and F). Maximum inhibition was observed against *Salmonella thyphimurium* (14.0mm) followed by *Staphylococcus aureus* and *Bacillus cereus.* The inhibitory activities of the purified extracts were also consistent with results determined on the isolates, as shown in Figure 1 (Panel A, B and C). *Lactococcus lactis* was commonly reported to show antagonism against closely related species or gram positive species (Choi *et al*. 2000; Moreno *et al*. 2000). The complex structure of gram negative bacteria hindered their action (Gong 2010). Up to now, only a few bacteriocins have been reported to inhibit gram negative bacteria and these include bozacin (Todorov *et al*. 2007), lacticin (NK24) (Lee & Paik 2001) and bacteriocin HV219 (Todorov *et al*. 2006). Antagonistic activity against gram negative bacteria is of particular interest because there are limited reports concerning LAB bacteriocin which is active against gram negative bacteria. Moreover, antagonistic activity against gram negative pathogens would enhance its use in food preservation and safety. Especially, in the control of foodborne pathogens, the effective use of nisin was only possible when it was used in combination with other methods such as high hydrostatic pressure (HHP) or in the presence of chemical such as EDTA (Sokołowska *et al*. 2013; Strevens *et al*. 1991).

**Figure 3:**
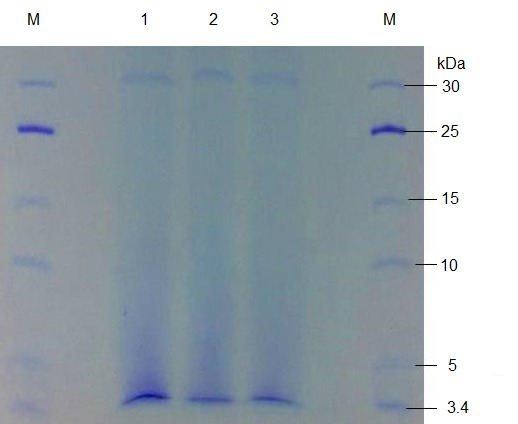
Purified bacteriocin fraction analysed using SDS-PAGE (14% gel). Lane M: Protein marker (PageRuler Low Range Unstained Protein Marker 3.4-100kDa, Fermentas), Lane 1, 2 and 3 contained samples of purified bacteriocin showing 3.4 kDa molecular weight bands.

Single step bacteriocin purification using chloroform resulted in a purified fraction at 74% recovery, as shown in the purification table (see Table 1). In SDS gel electrophoresis, purified fraction produced a single band of a size of about 3.4 kDa (Fig. 3). The low molecular weight range is in agreement with many bacteriocins reported for *Lactococci* especially nisin, the most extensively studied bacteriocin (de Vos *et al*. 1993). The small size bacteriocin is classified under Class I bacteriocin, a group called lantibiotic. Other than nisin, *Lactococci* were also reported to produce bacteriocin of slightly different molecular weight sizes. For example, Lactococcin BZ of 5.5 kDa molecular weight size was produced by *Lactococcus lactis* subsp. *lactis* BZ, isolated from Turkish boza (Şahİngİl *et al*. 2011). Other works had also reported on the low molecular weight bacteriocins isolated from *Lactococcus lactis,* ranging from 3 to 6 kDa (Aslam *et al*. 2012). Hence, being small in size, the bacteriocin from isolate A5 could also belong to nisin group, a lantibiotic.

The effect of different treatments on purified bacteriocin such temperature, EDTA, enzymes and detergents were shown on Table 2. The bacteriocin was found to be stable at 37°C and 65°C (for 15 min) but it showed partial stability at 100 °C by retaining at least 20% of its activity. Heat treatment at 121°C for 15 min resulted in a complete lost of activity. Usually, nisin or other bacteriocin produced from *Lactoccus* sp. showed to be heat stable. There was a complete inactivation in antimicrobial activity of bacteriocin with proteinase K treatment, as observed by Rashid *et al.* 2013. Sensitivities to proteolytic enzyme confirmed the proteinaceous characteristic of bacteriocin as this was also exhibited with nisin (Aslam *et al*. 2012). The bacteriocin activity was unaffected by alkaline pH, lysozyme or catalase treatments, a result of which similarly shown by *Lactobacillus plantarum* G_2_, (Šušković *et al*. 2011). Therefore, the antagonistic activity of bacteriocins from strain A5 was neither due to hydrogen peroxide nor organic acids, an observation supporting that the bacteriocin is protein in nature (Todorov *et al*. 2011).

The activity of extracted bacteriocin was significantly increased at 1% and 8% non-ionic detergents (Tween 20, Tween 80, Triton X-100) and an ionic detergent (SDS), but these activities were however suppressed upon treatment with EDTA. These trends were also observed by Aslam and co-workers (Aslam *et al*. 2012). However, in the case of lactococcin BZ, EDTA showed no effect (Şahİngİl *et al*. 2011). The addition of SDS to bacteriocin extract had enhanced its activity by 31%. The addition of anionic detergent had caused disaggregation of large molecules which resulted in a significant increase in antimicrobial effects (Malini & Savitha 2012).

## CONCLUSION

A bacteriocin producing LAB strain was isolated from the gut of Malaysian broad head catfish. The morphological, biochemical and rDNA sequence analysis showed that an isolate A5 resembled to that of *Lactococcus lactis*. This strain produced an antibacterial protein of 3.4 kDa molecular weight size. This bacteriocin was active against common Gram positive pathogens tested, such as *B. cereus* and *S. aureus*; and it also shared many common properties with other nisin-like bacteriocin such as being small in size, heat stable and protease sensitive. However, unlike many other *Lactococci* bacteriocins of Group 1, it also showed an antagonistic activity against gram negative pathogens, such as *S. thyphimurium.* This result showed that a nisin-like bacteriocin can have a broad spectrum of antagonism showing inhibition against gram negative pathogen. This strain and the bacteriocin it produced have great potential for use in applications such as catfish feeding, or any other feeding formulation in aquaculture. In addition to having properties related to that of established nisin, being active against gram negative bacteria could broaden its antimicrobial properties useful in food preservation and safety.

## Figures and Tables

**Table 1:** Purification table for purification of bacteriocin from the culture supernatant of strain A5.

Steps	Volume (ml)	Protein content (mg/ml)	Inhibitory activities (AU/ml)	Total activities (AU)	Total protein (mg)	Specific activities	Purification factor	Recovery %
Crude Cell free supernatant	30	0.60	71.2	2136.0	42.7	118.6	1.00	100.0
Chloroform precipitate sample	5	0.98	320.0	1600.0	313.6	326.5	2.75	74.9

**Table 2:** Sensitivities of bacteriocin extract from *Lactococcus lactis* strain A5 to temperature, enzymes and detergents.

Treatments	Zone of inhibitions (mm) ± SD
Temperature	
37°C	13.7 mm ± 0.58
65°C	13 mm ± 1
100°C	8.7 mm ± 0.58
121°C	No inhibition
Enzymes	
Proteinase K	No inhibition
Catalase	12.3 mm ± 0.58
Lysozyme	10.7 mm ± 0.58
Detergents (1%v/v)	
SDS	18 mm ± 0
Triton X-100	16 mm ± 0
Tween 20	16.3 mm ± 0.58
Tween 80	16 mm ± 0
EDTA	No inhibition
Detergents (8%v/v)	
SDS	16 mm ± 0
Triton X-100	17.7 mm ± 0.58
Tween 20	16.7 mm ± 0.58
Tween 80	16.3 mm ± 0.58
EDTA	No inhibition

*Note*: SD = Standard Deviation
